# A modification of a traditional Ethiopian maize store for solar powered ambient drying to reduce post-harvest losses

**DOI:** 10.1038/s41598-020-68485-2

**Published:** 2020-07-14

**Authors:** Franz Román, Chemeda Abedeta Garbaba, Christian Schellert, Oliver Hensel

**Affiliations:** 1 Department of Agricultural and Biosystems Engineering, University of Kassel, Nordbahnhofstr. 1a, 37213 Witzenhausen, Germany; 2 College of Agriculture and Veterinary Medicine, Jimma University, P. O. Box 307, Jimma, Ethiopia

**Keywords:** Engineering, Photovoltaics, Renewable energy

## Abstract

A *gombisa* is a traditional Ethiopian structure widely used for maize storage over several months. It lacks adequate ventilation for timely moisture removal, which promotes mold development and aflatoxin production. In this study, a traditional *gombisa* was compared to one modified by installing a solar powered fan to provide forced ambient air ventilation during daytime. Approximately 900 kg of moist ear maize were loaded into each structure and samples from selected locations were weighed periodically to monitor moisture loss. Temperature and relative humidity of ambient air and inside the maize bulk were continuously recorded. Significantly faster drying was achieved in the modified *gombisa*, where drying occurred from bottom to top. In the traditional store, drying was much faster at the surface, with drying rate declining sharply with increasing depth in the bulk due to more limited air exchange. Relative humidity in the bulk center of the traditional structure remained above 90% for more than 4 weeks while in the modified *gombisa* it decreased progressively from the beginning of the trial. The results are promising and the modifications simple to implement, with the potential to effectively reduce post-harvest losses of maize. Field tests in Ethiopia are recommended.

## Introduction

Maize is one of the most important staple foods in several African countries. In 2016 Ethiopia produced 7.84 Mt of maize and 8.11 Mt are estimated to have been produced in 2017^1^. It is predominantly produced by small scale farmers with over nine million smallholder households growing maize^2^ mostly for subsistence. It is the cheapest source of calories in Ethiopia and accounts for about 20% of the caloric intake of the population^3,4^.

It is widely acknowledged that one of the most effective ways to improve food security in Sub-Saharan Africa and other regions is to reduce quantity and quality losses during and after harvest. Preventing post-harvest losses is a major challenge for smallholder farmers in developing countries and conditions under which grain is stored ultimately impact their quality and quantity^5^. Estimated post-harvest losses of maize in Ethiopia range from 8.3 to 24%^6–8^, with drying and farm storage being two big contributing stages in the value chain^9^. Moreover, maize is very susceptible to aflatoxin contamination and this together with its high consumption in many African countries result in chronic exposure, often starting before birth^10^. Chronic exposure to aflatoxins has been linked to malnutrition, immunosuppression, and increased risk of liver cancer^10–12^. Acute aflatoxicosis has also been reported in Sub-Saharan Africa leading to fatalities^13–15^.

To minimize aflatoxin contamination and reduce losses, it is recommended to harvest shortly after physiological maturity is reached, when maize may contain up to 40% moisture (w.b.) and dry matter yield has reached its maximum^16^, followed by drying off the field. However, farmers often delay harvest for several weeks and let the crop dry in the field, exacerbating mold contamination and aflatoxin production due to increased insect infestation and damage, but still, a safe moisture content below 15% is seldom achieved^12^ (moisture content data reported as percentage are in wet basis, while data reported as index are in dry basis). Besides, farmers often lack the equipment necessary to establish whether their maize has attained the recommended moisture level long term storage^17^.

In hot, humid climates, the traditional methods used by farmers for drying grain rely on natural air movement to reduce moisture content to a safe level for storage. In addition, they may utilize the extra drying capacity gained by exposing the product to the sun^18^. For this, farmers typically use open storage structures to allow a substantial airflow^19^. In Ethiopia, maize is harvested and stored for gradual consumption until next season’s harvest, to fetch a better price and to keep the seed for the next planting season for subsistence farmers^20^. The *gombisa* is the most popular on-farm storage structure for ear maize in southwestern Ethiopia. It is usually an unplastered structure mostly made from bamboo, its roof is covered with thatched grass^21^. Similar cribs and granaries for maize storage are found in other east African countries like Kenya^22^ and Uganda^17^, and in Benin in West Africa where it is known as *ago*, for which poor aeration has already been noted^23^. When maize has not been properly dried, storage in traditional storage structures may lead to spoilage and mold development, especially in the interior of the bulk where exchange of air and moisture is strongly reduced. At the same time, the open nature of the walls and eventual roof leaks offer little protection from rain to rewet the crop^24^. Fleurat-Lessard^25^ cites a water activity limit for growth of aflatoxin-producing fungi and for aflatoxin production of 0.78 and 0.81 respectively. Thus, it should be aimed to bring relative humidity in the interstitial air of the maize bulk below these values by drying and aeration. Simple improvements to traditional storage structures could prove to be cost-efficient measures. A report by FAO^18^ recommends modifying traditional cribs and granaries, making them of a diameter or width of up to 1.5 m depending on the region´s climate and, in case of a rectangular structure, with the longer wall facing the prevailing wind and ensuring that the walls allow wind to flow through. Dubale et al.^26^ on the other hand, reported that *gombisas* in Ethiopia range in diameter from 1.48 m and up to 3.04 m. However, no experimental data were found in the literature on whether these dimensions consistently result in enough natural ventilation, especially of the inner zone of the contained maize bulk. A further modification to a traditional granary, proposed in this study, is to use a low power, photovoltaic driven fan to force ambient air during daytime hours when air temperature rises and relative humidity drops, expediting drying and potentially allowing the storage of maize harvested at higher moisture content shortly after reaching maturity.

In a preliminary study, a modified *gombisa* structure was built maintaining the overall appearance of an original structure. A detailed description of the construction of the store can be found in Garbaba et al.^20^. The structure has a cylindrical shape with a thatched roof on top. It has a diameter of 1.5 m and a height of 1.8 m. The main modification made compared to the original structures found in Ethiopia were a plenum chamber at the bottom, the installation of a brushless DC axial fan for forced ventilation and the inclusion of a plastic layer to the circular wall to make it air tight and therefore direct the air conveyed by the fan upwards through the maize bulk. The wall consists of three layers: an inner layer of steel reinforcement mesh, the middle layer of polyethylene tarpaulin, and an external layer of bamboo. A plastic sheet was also placed below the thatched roof to completely shed rain away. The intended bulk height of the structure was 1 m high, which corresponds to approximately 800–900 kg of ear maize.

Preliminary drying trials in 2016 showed that the mechanically ventilated *gombisa* could dry the maize from an initial moisture content of 0.22 to 0.14 kg kg^−1^ (d.b.) in 10–12 days under German mid and late summer weather^20^, which is roughly comparable with the conditions in Ethiopian midlands at harvest time. Schemminger et al.^27^ did a simulation study on ambient air drying of wheat under German weather conditions and of maize under Nairobi conditions. For the maize drying simulation, the size of the system corresponded to a 120-L plastic bin, as is common to find in Kenya for other purposes but fitted with a fan to force ambient air. They additionally modeled mold growth and aflatoxin production. Their results showed moisture content reduction from 21% to below 14% in little more than a day, but the continuous working of the fan could rewet the grain in the already dry layers. However, their study used shelled maize, which can dry considerably faster than ear maize due to its smaller particle size, thus impeding a direct comparison.

The preliminary trials on the modified *gombisa* did not compare those results with what would have occurred in a conventional structure without forced convection under the same weather conditions. Although common knowledge of ambient air crop drying indicates that a store equipped with forced convection should offer an advantage over a traditional structure, it is difficult to be sure about the extent of this improvement. This is because the provision of a closed wall in the modified structure (by means of the tarpaulin layer) to allow an effective upward airflow through the maize bulk eliminates the effect that wind and moisture diffusion might have in the conventional store. Therefore, the objective of this study was to do this comparison by constructing a second, this time conventional *gombisa* and perform a drying trial in both structures simultaneously.

## Materials and methods

### Study site

The present study was done at the experimental site for irrigation and solar technology of the Agricultural and Biosystems Engineering Department of the University of Kassel in Witzenhausen, Germany.

### *Gombisa* structures

The modified *gombisa* used by Garbaba et al.^20^ was used with some slight modifications. The tarpaulin mid layer which originally covered the entire height of the round wall (1.8 m), was cut 10 cm above the surface of the maize bulk. This was done to allow some exchange with the outside air during the night, which could be advantageous for the upper maize layer of the bulk since this is expected to be the last zone to dry. Also, a different brushless DC axial fan was used (ebm-papst, model 2214F/2TDHHO). The fan diameter is 200 mm, with a nominal voltage of 24 V and a nominal power of 48 W. Its maximum airflow rate at the rated voltage is 940 m^3^ h^−1^. The fan was connected directly to a set of two 30 W solar modules arranged in parallel. The matching of fan and solar panels was made so that the fan could start early in the morning and stop late in the afternoon, and that it was not strongly sensitive to solar radiation variation throughout the day, approaching its maximum airflow rate with relatively low levels of irradiance.

**Figure 1:**
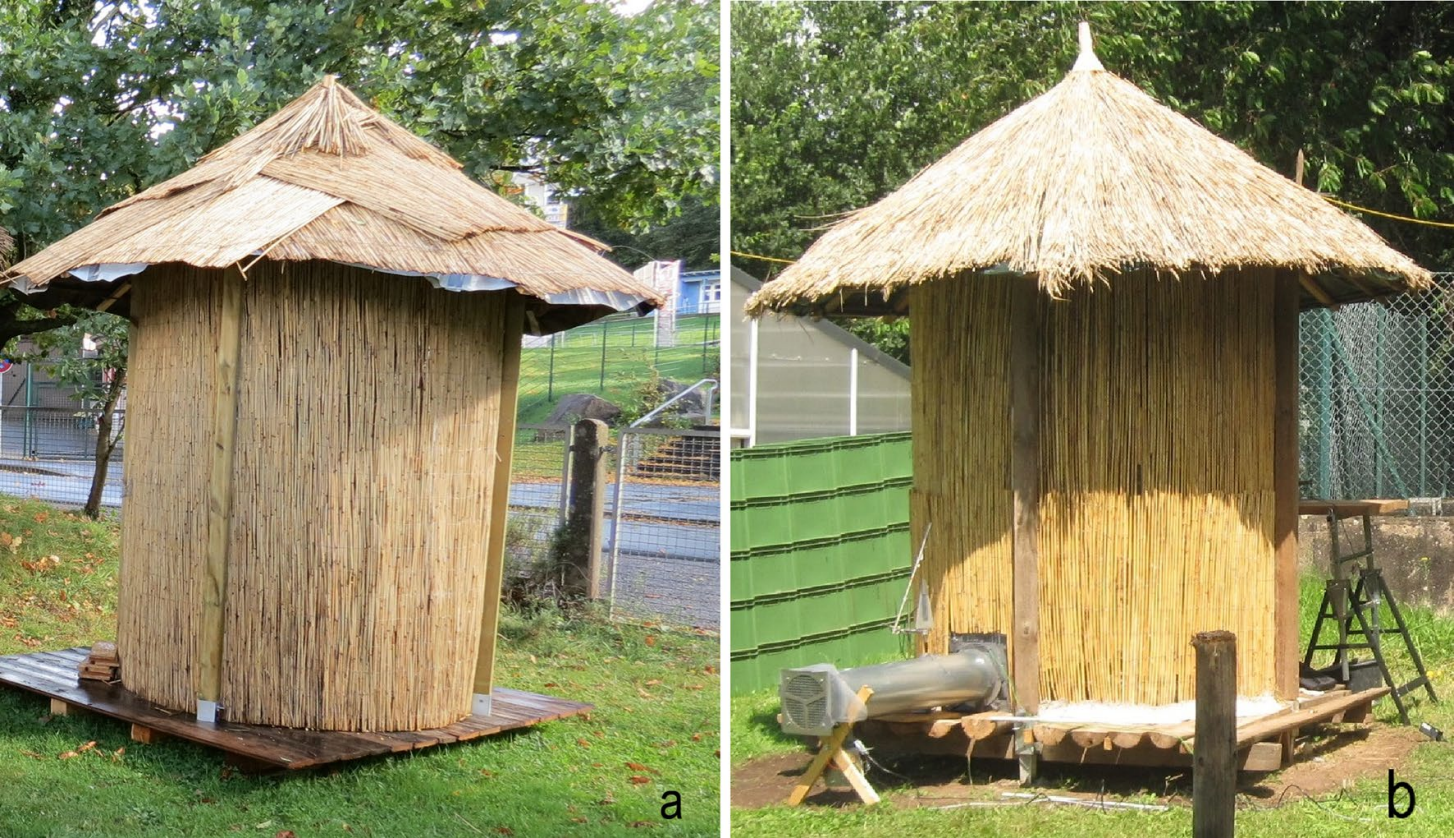
The two *gombisas* constructed: traditional type (**a**) and modified (**b**).

A second *gombisa* of the same dimensions and materials was built next to the first one, but resembling a conventional structure, that is, without an airtight wall nor forced convection, thus relying solely on wind and water vapor diffusion for drying (Fig. 1).

### Experimental procedure

The drying trial was done during late summer, from August 14 to September 27, 2018. The two stores were filled to a bulk height of one meter with ear maize. The maize had been harvested from the experimental site in Witzenhausen the year before in October, dried immediately and stored at a moisture content of around 0.13 kg kg^−1^. Before the drying trial the maize was rewetted for three hours in cold water. Afterwards the water was drained, and the maize left for 24 h to allow water at the ear surface to be absorbed or evaporate, and for moisture inside the ears to equalize. After rewetting the initial moisture content of the ears for the trial varied substantially (lowest initial moisture 0.415 kg kg^−1^, highest 0.824 kg kg^−1^). However, the variability at harvest in October of 2017, although lower, was still large (0.63–0.901 kg kg^−1^). The initial maize mass in each *gombisa* was around 900 kg.

**Figure 2:**
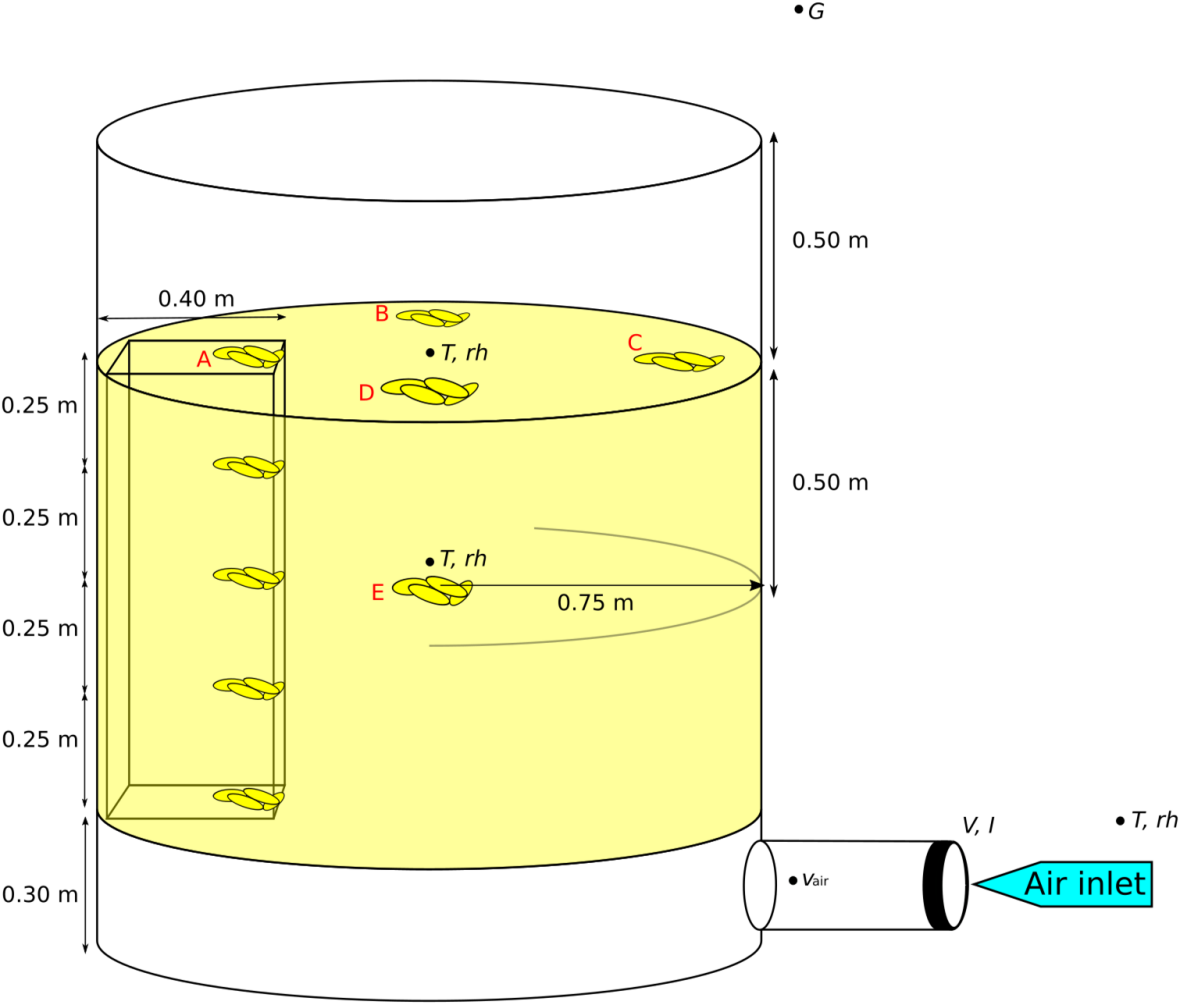
Dimensions of the *gombisas* and location of maize samples and measurement points. The duct and fan connected to the plenum chamber correspond to the modified *gombisa* only. All samples in the cage at different heights are represented by position A. The sampling position E is for the traditional *gombisa* only. *T *temperature, *rh *relative humidity, *G *global solar irradiance, *V *voltage, *I *current, *v*_*air*_ air velocity.

In bulk drying studies of shelled grain, it is possible to obtain samples from different depths with minimal disturbance of the bulk using grain samplers, which in the case of ear maize is not feasible. To be able to do this in this study, a metal cage with side dimensions 0.4 × 0.4 m and a height of 1 m was constructed for each *gombisa* and positioned inside it, adjacent to the wall (Fig. 2). The cages were filled with ear maize just like the rest of the store. Maize ear samples were weighed at the start of the trial, marked and placed in the cage at different heights in the bulk (0, 25, 50, 75 and 100 cm), four samples at each height. The samples were placed adjacent to the innermost wall of the cage, that is, almost 40 cm from the *gombisa* wall. Contrary to studies in which a different sample was taken each time from the same locations^28–30^, for most of the samples in this study the same units were retrieved and placed again in the bulk. This was decided due to the high variability expected in the initial moisture content of individual ears, which could have added noise to the results if different ears had been taken each time, as well as to avoid reducing the amount of maize in the stores. It was deemed preferable to follow the progress of specific units throughout the trial. Each time the samples were to be weighed, the maize in the cage was removed in order from top to bottom in four separate boxes (from 100 to 75 cm height, 75 to 50 cm, 50 to 25 cm and 25 to 0 cm). After samples were weighed, they and the rest of the ears were returned to the cage from bottom to top to preserve the original order. In addition to the samples in the cage, samples were also placed at the surface of the bulk at three other circumferential positions in a 90° angle to each other (positions B, C and D in Fig. 2). Weighing of the samples was done daily every morning with a Sartorius IC 34000 P scale from the beginning of the trial until day 9 of drying. Afterwards the weighing frequency was progressively reduced until the end of the trial, when the samples were dried at 105 °C for 72 h to determine their dry mass and calculate the moisture content at the moment of each weighing.

The aforementioned samples could be easily extracted for successive weighings due to their location in the cage or at the top of the bulk, and they provide useful information on the drying progress. However, it was also intended to monitor the moisture in those locations in the bulk with the least favorable conditions for drying. In the traditional *gombisa* this location is around the geometric center of the bulk, since this point is the farthest from the ambient air and it is increasingly difficult for wind to penetrate to such depth as the bulk size increases. Therefore, samples were taken from the center of the bulk at a height between 40 and 60 cm (position E in Fig. 2). For the modified *gombisa* the least favorable location should be at or near the top of the bulk, which is the layer constantly receiving the most humid air. Although samples at positions B, C and D might already represent the least favorable for drying, additional samples from 20 cm below these locations were also taken (not shown in Fig. 2 to avoid confusion). These locations in both stores are not easily accessible for samples to be removed and put back and doing so would have disturbed the bulk considerably. Thus, sampling from these positions was done only twice during the trial, different samples each time (they were taken from the bulk but not put back) and their moisture content at the time determined.

During the drying trial measurements of global horizontal irradiance were made with a Kipp and Zonen SP-Lite silicon pyranometer with a sensitivity of 79 µV/W m^−2^. The pyranometer was placed close to the solar modules in the roof of a neighboring building, free from shade. Fan current and voltage were also measured. For current, a 10 mΩ ± 0.1% shunt resistor was used. To measure airflow in the modified *gombisa*, an Airflow TA5 hot wire anemometer with a full scale accuracy of ± 1% (full scale of 15 m s^−1^) was installed in the air duct one meter downstream from the fan, with the sensing wire placed at the center of the duct cross-section. To calculate the airflow rate in the system from a single measurement point, the guideline VDI/VDE 2640 Part 3^31^ was used, which details the procedures to determine the flow rate of gases in ducts of circular, annular and rectangular cross sections by means of grid measurements. Measurements of irradiance, voltage, current and air velocity were recorded every minute by a data acquisition unit (Agilent 34970a). Temperature and relative humidity of ambient air as well as the conditions inside the stores were recorded using Testo 174H data loggers (± 0.5 °C, ± 3% r.h.) at five-minute intervals. For each *gombisa* a device was placed at the center of the bulk surface, while a second was placed also at the center but 50 cm below the surface (50 cm bulk height), which is the centroid of the bulk (Fig. 2).

### Statistical analysis

To test for normality and equal variances among groups the Shapiro–Wilk test and Bartlett test were used, respectively. For comparing two groups the *t* test was used. More than two groups were compared with one-way analysis of variance (ANOVA) followed by multiple comparisons *t* test with Bonferroni correction. The statistical analysis was performed in R.

## Results and discussion

**Figure 3:**
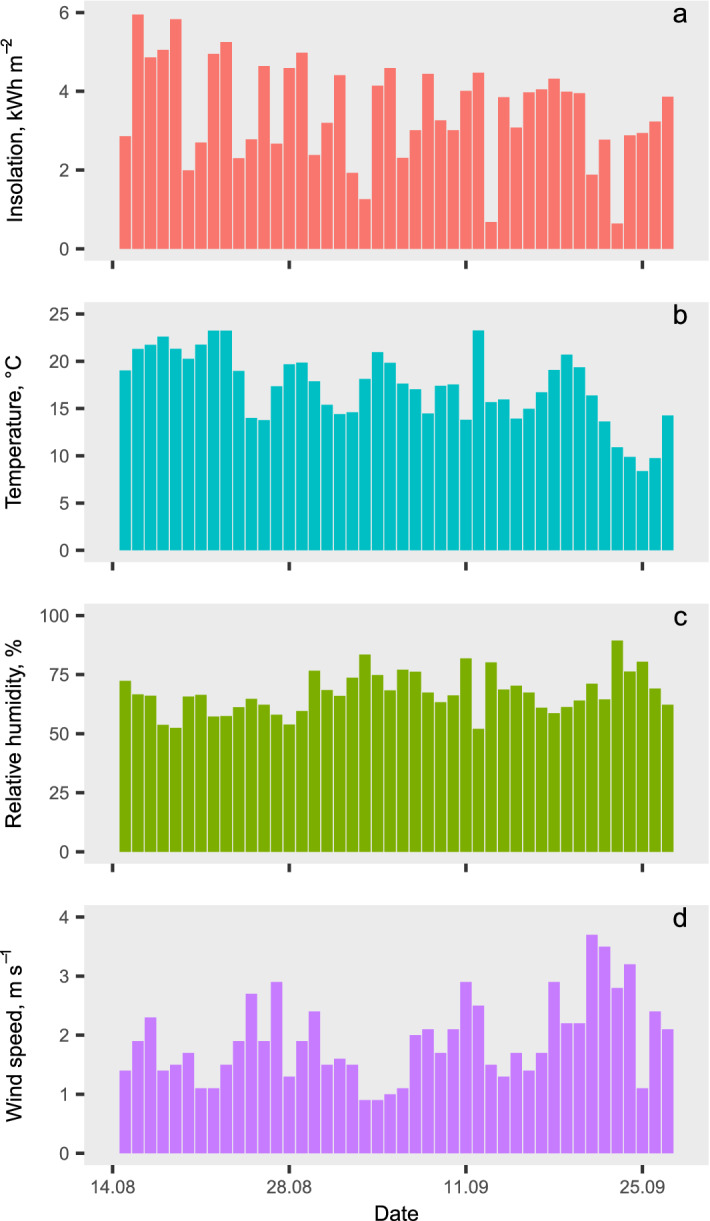
Daily global insolation and daily average temperature, relative humidity, and wind speed in the study area during the trial.

The climatic conditions during the trial were mostly favorable for natural air drying. Most days had ample solar radiation approaching or surpassing 700 W m^−2^ during midday, and with more than 50% of the days reaching at least 25 °C, several days with maximum temperatures above 30 °C. Daytime relative humidity was well below 60%, with minimums often below 40%. Due to predominantly clear sky, night temperatures often approached or fell below 10 °C bringing relative humidity above 90%. Figure 3 shows the total daily insolation and the daily average ambient temperature and relative humidity from own measurements at the study site, as well as the daily average wind speed from a nearby weather station. The insolation and ambient temperature presented the normal downward trend as the solar elevation and daylight hours decreased from mid-August to late September, whereas the average relative humidity increased slightly. Wind speed registered higher values towards the end of the trial.

**Figure 4:**
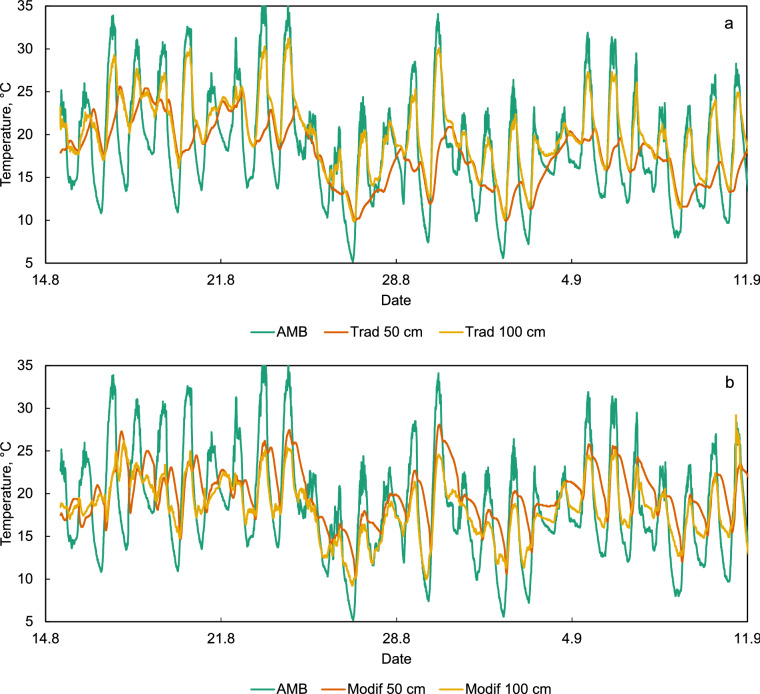
Temperature of ambient air, air inside the maize bulk (50 cm) and in the bulk surface (100 cm) in the traditional (**a**) and modified (**b**) *gombisas.*

Figure 4 shows the temperature curves of ambient air and of the conditions inside both stores during the first 4 weeks of trial. In the traditional store (Fig. 4a) the daily oscillations at the bulk surface (100 cm) appear to be in phase with the ambient temperature, since the bulk surface receives outside air directly due to wind. In the middle of the bulk there is a slight shift to the right in peaks and troughs. This is explained by the self-heating that occurs in wet grain even as the temperature of the surroundings falls sharply at night, helped by the thermal insulating nature of the bulk. In the case of the modified *gombisa* (Fig. 4b) the diurnal temperature at the grain surface does not reach values as high as in the traditional structure, since cold and humid air reaches this zone from below. It can also be seen that the daily temperature oscillations in the middle of the bulk are also initially shifted relative to the ambient conditions, but this shift disappears gradually as drying progresses and moisture content in the bulk falls, limiting self-heating during the night. The temperature shift is also initially present at the bulk surface, contrary to the traditional *gombisa*, likely due to the tarpaulin layer in the wall partly blocking the entry of outside air.

**Figure 5:**
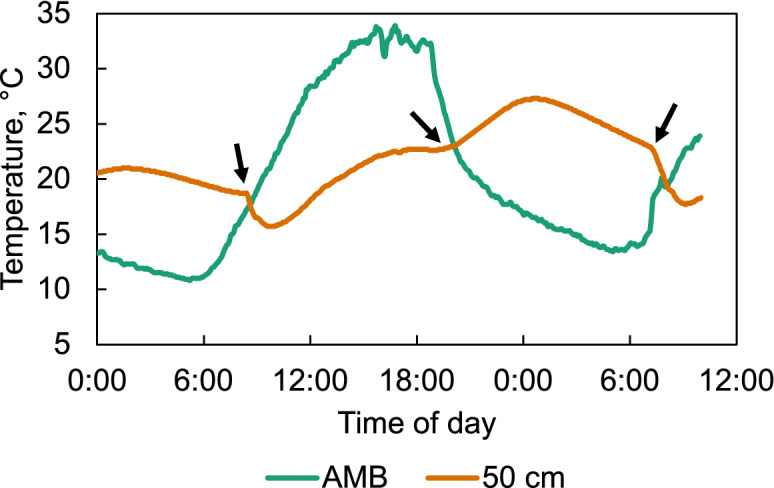
Ambient temperature and temperature at mid-height in the modified *gombisa* during August 16 and half day of August 17. The arrows indicate the moments when the solar driven fan stopped or started.

The starting and stopping of the fan according to the available solar radiation can be discerned from the temperature curve at mid height of the bulk as shown in Fig. 5 for August 16 and 17. The first and third arrows mark sharp temperature changes when the fan starts in the morning, evaporative cooling resumes, and temperature drops below ambient. The arrow in the middle marks the moment when the fan stops completely in the evening and no more evaporative cooling occurs, so that self-heating of the grain allows it to increase the inner temperature above ambient.

**Figure 6:**
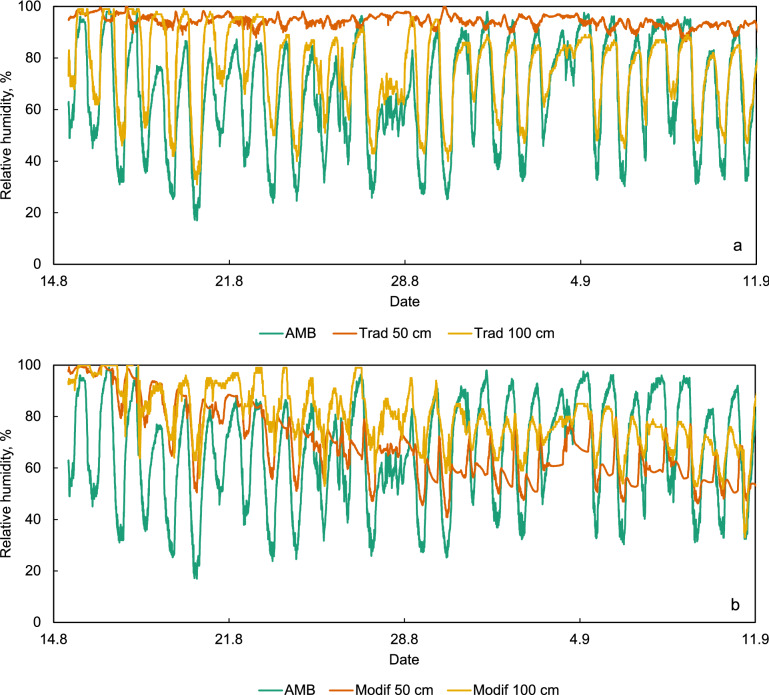
Relative humidity of ambient air, air inside the maize bulk (50 cm) and in the bulk surface (100 cm) in the traditional (**a**) and modified (**b**) *gombisas*.

The ambient relative humidity also oscillated daily, falling during the day and raising at night (Fig. 6). The humidity at the surface of the maize bulk in the traditional *gombisa* follows the ambient conditions more closely than in the modified, dropping more during the day. At night, during the initial stages of the trial, it rises in both structures above the ambient conditions due to the inner climate formed by the wet maize. This is slightly more pronounced in the modified *gombisa*. As the drying continues, the relative humidity at the surface remains below the ambient value at night in both structures, again more markedly in the modified *gombisa*.

More important and telling is the difference in relative humidity inside the maize bulk (at 50 cm). Placed as a bulk, a moist product creates its own internal climate in terms of temperature and relative humidity of the interstitial air. A traditional *gombisa* with its permeable wall relies exclusively on wind and diffusion to remove moisture from maize. However, relative humidity in the center of the bulk remained above 90% for prolonged periods of time, even during daytime when ambient humidity often fell below 40%, indicating slow drying and conditions favorable for mold development and aflatoxin production. Even though ear maize offers relatively low resistance to airflow in a system with forced convection compared to shelled grain, in a store of this diameter wind by itself does not seem able to penetrate to the center of the bulk sufficiently to reduce relative humidity even briefly to remove moisture and facilitate drying. In the modified store, the gradual diurnal reduction of the gap between the ambient relative humidity and that in the middle of the bulk as the days go by clearly depicts the drying progress, and r.h. gradually reduces to values below 70% and then well below 60%.

The relative humidity curve at mid height of the bulk in the modified *gombisa* also clearly shows the influence of the fan shutting on and off, especially after the second week of drying. As ambient humidity rises late in the afternoon, the humidity inside the bulk rises accordingly until the fan stops due to insufficient solar radiation. Here relative humidity declines sharply below ambient levels towards the value dictated by the current moisture content of the maize in that location. The next morning, when the fan starts the humidity inside the bulk rises suddenly to follow again the trend in the ambient conditions.

Since the modified *gombisa* has forced convection of ambient air, the curves of Figs. 4b and 6b can be seen as depicting the change in air conditions from bottom to top of the bulk due to the drying process during daytime, when the fan is working.

**Figure 7:**
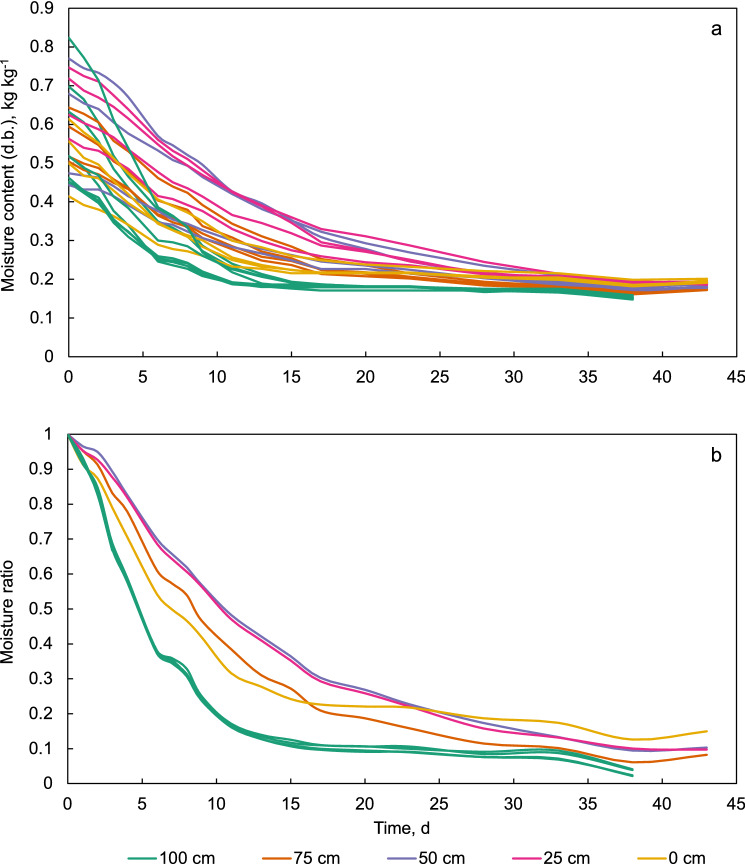
Drying curves of individual maize samples as moisture content (**a**) and average moisture ratio (**b**) in the traditional *gombisa*.

Figure 7a shows the individual curves of moisture content versus drying time of all maize samples in the cage in the traditional *gombisa*. Additionally, the curves of some of the samples at the bulk surface outside the cage (positions B, C and D in Fig. 2) are shown belonging to the “100 cm” label. The curves are grouped by height in the bulk with different colors. The spread of initial moisture content of the samples is evident. Although this could appear to be due to the rewetting prior to the start of the trial, the variability in moisture was almost as wide at harvest, and with even higher moisture contents in general, as already mentioned. Figure 7b shows the drying curves more succinctly as the average of each sampling position, but in terms of the dimensionless moisture ratio (MR). For the moisture ratio, an equilibrium moisture of 0.14 kg kg^−1^ was used, since this is slightly below the lowest moisture content achieved by any of the samples. The maize at the bulk surface dried substantially faster than the rest. This was expected since the porous wall of this structure allowed wind to flow into the air space above the surface of the maize bulk almost unimpeded, providing constant fresh air day and night. Since this *gombisa* also had a false floor 30 cm above the ground, a similar effect was expected for the maize at the bottom (0 cm height), but this was barely the case during the first weeks of the trial and afterwards the drying rate at that location even fell below that of the inner maize layers (25, 50 and 75 cm). This could have been due a higher relative humidity closer to ground which stalled drying compared to higher locations in the bulk.

**Figure 8:**
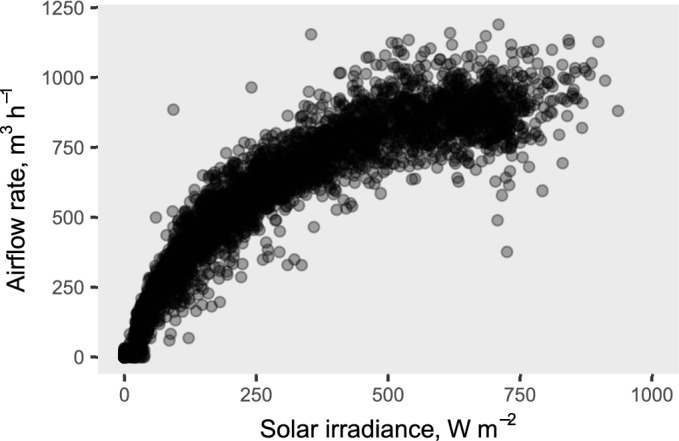
Coupled measurements of airflow rate in the modified *gombisa* and solar irradiance during the drying trial.

Airflow conveyed by the fan in the modified *gombisa* was recorded until September 11, when the drying in this store was already considered to be completed. The fan started every morning usually with a horizontal solar radiation below 40 W m^−2^ and stopped only when it fell again below 30 W m^−2^ in the evenings. Figure 8 shows all the instantaneous measurements of airflow rate versus solar irradiance. In average, with 400 W m^−2^ the airflow rate was already 83% of that at 800 W m^−2^, resulting in low fluctuations in airflow rate between medium and high levels of solar irradiance.

**Figure 9:**
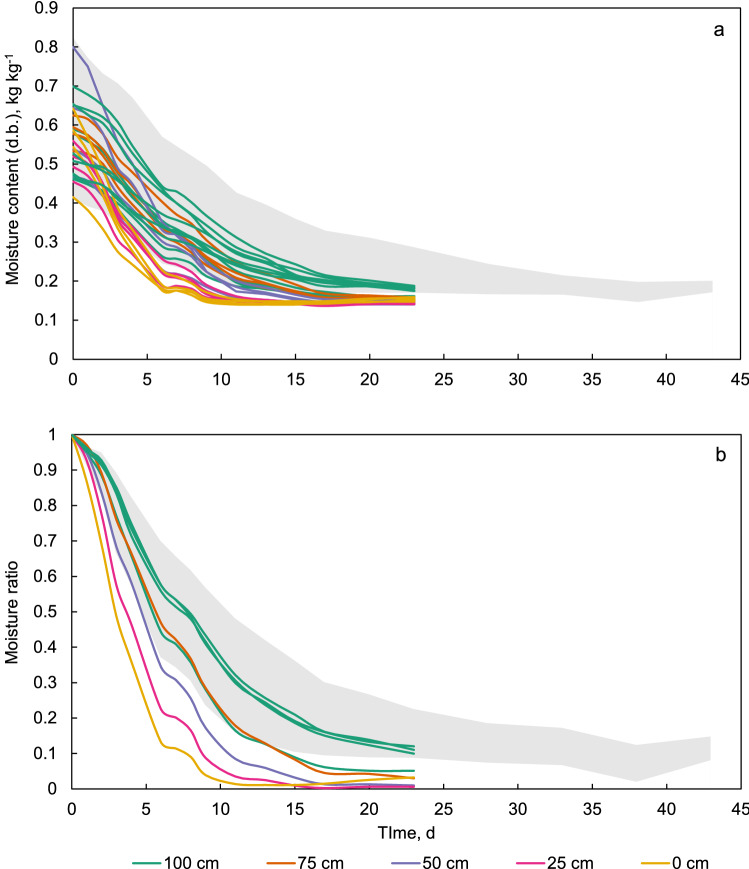
Drying curves of individual maize samples as moisture content (**a**) and average moisture ratio (**b**) in the modified *gombisa*.

Figure 9a shows the drying curves of the individual maize samples in the modified *gombisa*. As in Fig. 7a, the curves for some of the samples at the bulk surface outside the cage are included under the label “100 cm”. For comparison purposes, the region spanning all the drying curves of the samples in the traditional store is also depicted in the figure as a grey area. The weighing of maize samples in this structure was done until the 23rd day of the trial, when weight loss had stopped in the samples in the cage. However, the remaining maize was left in the *gombisa* and the fan continued running until the complete trial was ended. As in Fig. 7b, Fig. 9b shows the average drying curves for each sampling location in terms of the moisture ratio. Here, drying clearly occurs from bottom to top following the air direction, as was expected from deep bed drying theory. Drying until equilibrium with environmental conditions was accomplished within 11 days at the bottom of the bulk (0 cm height) while the layers above took gradually longer.

Also evident from Fig. 9a is that the maize in the modified store seemed to reach a lower equilibrium moisture with the drying air compared to the traditional structure. For example, in the 23rd day, some of the maize samples had attained a moisture content close to 0.14 kg kg^−1^ for several days already, and half of the samples in the cage were below 0.15 kg kg^−1^. On the other hand, at the same time in the traditional *gombisa* the fastest drying samples also seemed to be in equilibrium with their surroundings (the curves are flat) already for some days, but this equilibrium was above 0.17 kg kg^−1^.

In Fig. 9, a group of curves from the bulk surface (100 cm height) can be distinguished from the others as drying slower. These correspond to the samples outside the cage (positions B, C and D in Fig. 2). The fact that this was consistently the case hints to a slightly higher airflow rate in the cage than in the rest of the bulk. This might have been caused by a near-wall channeling effect caused by the cage itself, which allows higher airflow near the cage walls, as well as due to the location of the cage opposite the fan inlet, since it has been often observed in the literature that this region usually receives slightly higher air velocities through the bulk^32^. Focusing on the samples inside the cage however, it seems from Fig. 9b that in the modified *gombisa* the samples at 75 and 100 cm height dried at practically the same rate. One would expect the samples at 75 cm height to dry slightly faster, but since the upper wall of this store was open (no tarpaulin sheet), the maize at the bulk surface was also exposed to ambient conditions especially when the fan was off, allowing further moisture removal during the night from this layer.

To test whether the difference in drying depending on position in the bulk was statistically significant, the drying curve of each sample ear (in terms of MR) in the metal cages of each *gombisa* was characterized by its average drying rate from the start of the experiment until the day in which the curves of the fastest sampling position went flat. This was considered day 17 in the traditional *gombisa* (when the MR curves at 100 cm height went flat, Fig. 7), and day 11 in the modified (when the MR curves at 0 cm height went flat, Fig. 9). Table 1 shows the average, minimum and maximum drying rate for the traditional and modified stores at each position). It can be reasonably assumed from the nature of the data that the drying rate of the samples is normally distributed, and the Shapiro–Wilk normality test confirmed this.

**Table 1:** Summary of drying rates depending of height in the bulk in both *gombisas*, until day 17 in the traditional, and day 11 in the modified.

Sample height, m	n	Drying rate (day^−1^)		
		Traditional		
		Average	Min	Max
100	4	0.05314	0.05255	0.05384
75	4	0.04650	0.04505	0.04792
50	4	0.04102	0.03888	0.04305
25	4	0.04160	0.03953	0.04372
0	4	0.04551	0.04260	0.04807

Sample height, m	n	Drying rate (day^−1^)		
		Modified		
		Average	Min	Max
100	4	0.07597	0.07309	0.07947
75	4	0.07470	0.07066	0.07670
50	4	0.08324	0.07996	0.08628
25	4	0.08773	0.08581	0.08915
0	4	0.08965	0.08840	0.09086

Analysis of variance showed a significant difference in drying rate depending on height in both stores (p-values of 2.413 × 10^–7^ and 1.107 × 10^–6^ for traditional and modified *gombisas* respectively). Multiple comparison *t* tests with Bonferroni correction showed mostly significant differences (alpha = 0.05) in drying rate at the different heights for the traditional *gombisa*, with the exception of the pairs 0 and 75 cm, and 25 and 50 cm (Table 2). In the modified store drying rates differed except for the pairs 0 and 25 cm, 25 and 50 cm, and 75 and 100 cm.

**Table 2:** Bonferroni corrected p-values in multiple comparison *t* tests for the drying rate at different heights in the maize bulk in the traditional and modified *gombisas*.

	Traditional			
	0 cm	25 cm	50 cm	75 cm
25 cm	0.048	–	–	–
50 cm	0.0176	1.000	–	–
75 cm	1.000	0.0087	0.0032	–
100 cm	0.0001	6.9 × 10^–7^	3.6 × 10^–7^	0.0005

	Modified			
	0 cm	25 cm	50 cm	75 cm
25 cm	1.0000	–	–	–
50 cm	0.0338	0.2770	–	–
75 cm	7.3 × 10^–6^	3.8 × 10^–5^	0.0033	–
100 cm	2.1 × 10^–5^	0.0001	0.0129	1.0000

**Figure 10:**
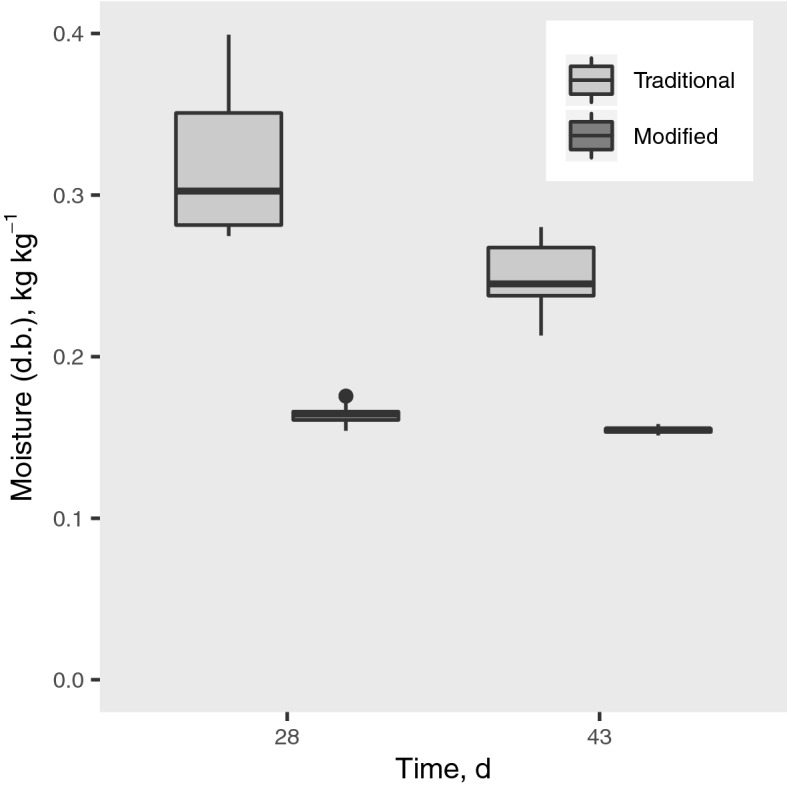
Moisture content in the slowest drying zone of the bulks at days 28 and 43 in both *gombisas*.

The drying curves and the calculated drying rates already demonstrate a considerable advantage of the modified store. However, the situation is even more negative in the traditional store if the least favorable location for drying (as described in the experimental procedure) is considered (Fig. 10). Maize in this region was still far from a safe moisture content after 4 weeks, and moisture varied strongly between samples, in a range from 0.275 to 0.399 kg kg^−1^. Even after 6 weeks moisture was still high and highly variable (0.213–0.281 kg kg^−1^). In the modified store at 4 weeks moisture was already very uniform between samples (0.1541–0.1757 kg kg^−1^) and almost in equilibrium with average ambient conditions. Unequal variance two-sided *t* tests confirmed significant differences between the stores in their slowest drying zones, with p-values of 2.594 × 10^–5^ and 2.256 × 10^–7^ for 28 and 43 days respectively.

According to Dubale et al.^26^, *gombisas* usually have a diameter between 1.48 and 3.04 m. Therefore, the size of the structures investigated in this study lie in the lower end; the situation in the center of bigger traditional stores can only worsen, probably disproportionately. However, once a *gombisa* is fitted with a fan of adequate power, the diameter of the structure would have little influence in performance.

The course of forced convection drying in a deep bed is strongly dependent on the air temperature and airflow rate employed. In the present study, the combination of large particle size, limited bed height and low air temperature allowed drying in the upper maize layer to start from the beginning of the trial. After 23 days of intermittent ventilation, the maize could be dried from an average moisture of 0.552 to 0.155 kg kg^−1^. In the preliminary study on the modified *gombisa*^20^ drying from 0.22 to 0.14 kg kg^−1^ took 12 days in mid-September. A previous study on natural air drying of ear maize in North America was done during the fall in an industrial dryer with a bed depth of 4.4 m^33^. In this case the top layer remained at the initial moisture for several days and drying was completed in 31 days from 0.314 to 0.199 kg kg^−1^. In another study using beds 2.1 to 3.4 m deep, continuous ventilation at temperatures between 35 and 46 °C and airflow reversal, ear maize could be dried from 0.47 to 0.136 kg kg^−1^ in 3–4 days.

The forced airflow in the modified store accelerates drying but also serves to even out the difference between ears of high and low initial moisture content, since drying rate is proportional to the difference between current and equilibrium moisture. The size of fan and solar panels used in this study were selected to provide a superficial air velocity of 0.1–0.15 m/s during most of daytime. Bigger fan and panel sizes would increase airflow and drying rate further, as well as reduce variations between the top of the bulk and the bottom. Moreover, a small solar air heater made of accessible, inexpensive materials could further improve performance by increasing the ambient temperature a few degrees. The installation of a low power DC fan directly coupled to a solar array is simple and needs practically no maintenance. Thus, the modifications presented can be useful to allow Ethiopian farmers to dry maize harvested soon after physiological maturity, when moisture content is still high, before insect damage and mold infection in the field is widespread and carried to storage. Although the present study was performed under German late summer conditions, these are roughly comparable to Ethiopian conditions in the mid-lands during maize harvest. The results are encouraging and merit further tests in different maize-growing regions of Ethiopia, which are being planned.

## Data Availability

The datasets generated during and/or analyzed during the current study are available from the corresponding author on reasonable request.
